# Improvement in the Method of Mounting Artificial Teeth

**Published:** 1850-10

**Authors:** 


### IMPROVEMENT IN THE METHOD OF MOUNTING ARTIFICIAL TEETH.

For several years past a few dentists among our acquaintance have been in the practice of soldering their artificial teeth, for entire lower sets, to the gold plates with pure tin, using the tinman's soldering iron instead of the blow-pipe. The manner of proceeding is as follows.

First strike up, in the usual manner, a very thin gold plate (No. 30 or 31, will answer) to fit the jaw. When this is done, place the wax upon it and cut it to the right curve and the proper height for the length of the teeth. The teeth are then to be selected and put round upon the wax in the proper position for use; but it does not matter whether, or not, they come down to the plate, provided all that part of them which is exposed to view, when in the mouth, is right, as all below will be filled with tin when the process is completed. Plaster and sand is now to be put on the outside of the teeth and plate, in the same manner as though they were to be soldered in the usual way. When this is done the wax may be cut away, the teeth removed from the plaster and a thin gold back put upon them. In backing them it will be necessary to bend the platina wires together, over the gold, with a common pair of pliers. The backs may now be soldered to the plate forming one solid mass of tin as high as the wires and imitating as nearly as possible the form of alveolus which has been absorbed. When this is done the plaster may be taken away and as much tin put upon the front as will restore what has been lost by absorption of gum and alveolar process.

When the piece is properly trimmed and burnished it makes a very strong and natural set of teeth, while the additional weight given to it by the tin keeps it in place better than those made in the ordinary way. Some use silver plate instead of gold and gild the whole by the galvanic process, and we can see no reason why this metal should not answer just as well as gold. We have put in several temporary sets in the above manner, on gold and all have done remarkably well, giving entire satisfaction. This plan of mounting teeth was first practiced, we believe, by Mr. Royce, about eight years since and has been used by him in very many cases, as he alleges, with perfect success.

Mr. George E. Hawes has lately made an improvement upon the above plan by means of which he dispenses with all metalic castings and plates of every kind, using only the pure tin and the teeth. His plan is, after the first cast is procured, which should be made of plaster with a large proportion of sand, to fit to it a piece of tin foil, or plate, as thick as can well be rubbed down to it with a burnisher, and as large as a gold plate would have to be. The wax is then put upon this tin plate and trimmed to the proper curve and height as in ordinary practice. Next, the teeth are to be placed upon the wax and when properly arranged a strip of wax is put round the bottom of the front side of the teeth and plate. This wax, and that on the backs of the teeth, is then to be carved to represent the natural gums or so as to form a smooth ridge as high as is desirable. Care must be taken to select such teeth as have their platina pins low, so that they may remain embedded in the wax.

When this process is completed, the whole is to be placed upon the plaster and sand cast and more plaster and sand poured over it so as to cover with a thick mass the whole of the wax and the teeth. After the plaster has thoroughly hardened the casts may be parted, and the tin plate and all the wax taken away, and the platina wires, and those parts of the teeth exposed washed with muriate of tin. A hole to pour the melted tin into must now be made at one end of the set and another on the other side for the air to escape from. When completed thus far it is ready for the pouring, and to insure perfect success the castings should be securely bound together and the whole mass heated to the temperature of melted tin.

Sets of teeth made in this way and having the casting thoroughly gilded are much handsomer and more natural in their form than those which have the long teeth and gold backs; they are also stronger, as they are protected both front and back, can be made for one half the expense of the ordinary sets on heavy gold plates, and, judging from the little experience which we have had in making and testing them, as well as the testimony of Mr. Hawes, are equal in every respect, if not superior to those mounted upon gold backs.—*Dental Recorder.*

